# Does Every Calculation Formula Fit for All Types of Intraocular Lenses? Optimization of Constants for Tecnis ZA9003 and ZCB00 Is Necessary

**DOI:** 10.3390/medicina57040319

**Published:** 2021-03-30

**Authors:** Ivajlo Popov, Veronika Popova, Juraj Sekac, Vladimir Krasnik

**Affiliations:** 1 Department of Ophthalmology, Faculty of Medicine, Comenius University, 821 01 Bratislava, Slovakia; ivajlo.popov@gmail.com (I.P.); sekac.juraj@gmail.com (J.S.); 2 Department of Pediatric Ophthalmology, Faculty of Medicine, The National Institute of Children’s Diseases, Comenius University, 831 01 Bratislava, Slovakia; veronika.labuzova@gmail.com

**Keywords:** intraocular lens, power calculation formula, biometry, cataract, surgery

## Abstract

*Background and Objectives*: To evaluate the performance of intraocular lenses (IOLs) using power calculation formulas on different types of IOL. *Materials and Methods*: 120 eyes and four IOL types (BioLine Yellow Accurate Aspheric IOL (i-Medical), TECNIS ZCB00, TECNIS ZA9003 (Johnson & Johnson) (3-piece IOL) and Softec HD (Lenstec)) were analyzed. The performance of Haigis, Barret Universal II and SKR-II formulas were compared between IOL types. The mean prediction error (ME) and mean absolute prediction error (MAE) were analyzed. *Results*: The overall percentage of eyes predicted within ±0.25 diopters (D) was 40.8% for Barret; 39.2% Haigis and 31.7% for SRK-II. Barret and Haigis had a significantly lower MAE than SRK-II (*p* < 0.05). The results differed among IOL types. The largest portion of eyes predicted within ±0.25 D was with the Barret formula in ZCB00 (33.3%) and ZA9003 (43.3%). Haigis was the most accurate in Softec HD (50%) and SRK-II in Biolline Yellow IOL (50%). ZCB00 showed a clinically significant hypermetropic ME compared to other IOLs. *Conclusions*: In general, Barret formulas had the best performance as a universal formula. However, the formula should be chosen according to the type of IOL in order to obtain the best results. Constant optimizations are necessary for the Tecnis IOL ZCB00 and ZA9003, as all of the analyzed formulas achieved a clinically significant poor performance in this type of IOL. ZCB00 also showed a hypermetropic shift in ME in all the formulas.

## 1. Introduction

On 29 November 1949, Sir Harold Ridley for the first time implanted an artificial intraocular lens (IOL) into the human eye. The postoperative refraction was −21 diopters (D) in the operated eye [1]. Subsequently, new methods were sought to improve the estimation of postoperative refraction.

The precise biometry of the eye before surgery has grown in its importance. New formulas which used these parameters for the calculation of the estimated postoperative refraction have been developed [2]. As patients’ postoperative expectation rapidly increased, IOL calculation has come into focus in recent years [2]. Initially, biometers used ultrasound to measure eye dimensions [3], but optical biometers were introduced in the late 1990s [4]. The reproducibility of measurements and convenience were greatly improved by optical biometers [5]. Improvements also continued in the IOL calculation formulas, such that, the theoretical formulas were continuously replaced or adjusted by newer formulas such as SRK II [6] and by modern formulas used currently. There are some recommended formulas used depending on the axis length [7], but some of the newer formulas perform well across all the axial lengths [8].

There are many designs of IOLs that can determine postoperative lens position inside the capsular bag and have a great influence on the IOL performance. In this study, we compared the performance of the IOL calculation formulas in different types of popular IOLs that are not commonly analyzed in published studies. We compared some of the newer commonly used IOL formulas (Haigis and Barret Universal II) in contrast to one of the oldest formulas (SRK-II). 

## 2. Materials and Methods

The design was a retrospective consecutive case series, which was studied at a University Hospital (Department of Ophthalmology, University Hospital Ruzinov in Bratislava, Slovakia) only. The aim of this study was to analyze how different IOL calculation formulas performed in IOLs with different designs and materials. This study adhered to the tenets of the Declaration of Helsinki.

All analyzed subjects underwent uncomplicated cataract surgery between January and September 2017. The exclusion criteria were keratoconus, preoperative or postoperative astigmatism of more than 3.0 diopters (D), previous refractive surgery, complicated cataract surgery, or a history of vitrectomy. Surgeries were performed by multiple surgeons but with a similar technique. Phacoemulsification was performed through a 2.75 mm superior incision under topical anesthesia. Before surgery, an eye biometry and IOL calculation were carried out with an OLCR biometer Lenstar LS900 with built-in IOL calculator. No formula constants optimization was performed for any surgeon. If both eyes underwent surgery in the hospital, only the right eye was included. Otherwise, only the operated eye was included. Eyes measured with ultrasound biometry were excluded. Postoperative refraction was measured at least two weeks after surgery with automatic kerato-refractometer. A two-week period was chosen to eliminate the possible effect of postoperative corneal edema, wound healing or other factors which could affect the measurements. Preoperative and postoperative refraction were displayed as spherical equivalents. Subjects were divided into groups according to implanted IOLs. Intraocular lenses used in the sample were the BioLine Yellow Accurate Aspheric IOL (i-Medical, Mannheim, Germany), TECNIS ZCB00, TECNIS ZA9003 (Johnson & Johnson, New Brunswick, NJ, USA) (3-piece IOL) and Softec HD (Lenstec, St Petersburg, FL, USA). Eyes with axial lengths from 21 to 26 mm were included. Extremely short and long eyes were excluded. The mean error (ME) of prediction for each IOL formula was calculated. The ME was calculated as the difference between the postoperative refraction subtracted from the estimated refraction using the formula. The mean absolute error (MAE) for each IOL formula was also calculated as the absolute value of ME.

### Statistical Analysis

The patient’s data were analyzed by means of descriptive and inferential statistics. Continuous or interval-scaled variables were first checked for normality and outliers through visual inspection. Descriptive analyses were performed on all selected variables. The differences in the MAE and ME between dependent variables were assessed using the Friedman test. In the event of a significant result, the t-test for normally distributed values and the post hoc Wilcoxon test for non-normally distributed values was used. In the case of independent variables, the parametric ANOVA or nonparametric Kruskal–Wallis test was used. All tests were adjusted with Bonferroni correction. *p* value after adjustment (Bonferroni) less than 0.05 was considered statistically significant.

## 3. Results

120 patients (120 eyes) were analyzed. Each IOL group consists of 30 eyes. The demographic data of the sample are shown in Table 1. 

The longer axial length in ZA9003 group was statistically significant (*p* < 0.05). The age was significantly higher in the Softec HDM group compared to the ZA9003 and ZCB00 group (*p* < 0.05) and in the BY group compared to the ZA9003 group (*p* < 0.05). Differences in other demographic parameters were not significant (*p* > 0.05). In the linear regression model, the interaction of the axial length and the age on the ME or MAE was not statistically significant (*p* > 0.05). The correlation between the AL and ME or MAE of the whole sample was weak and statistically insignificant (Pearson for the ME = −0,124; *p* > 0.05; for the MAE = −0,106, *p* > 0,05). The results of the ME and MAE for the whole sample and each IOL type are shown in Table 2, separately.

Table 2 shows that the Barret Universal II formula (hereafter simply referred to as the “Barrett”) had the lowest ME and MAE (the MAE was same in the Haigis formula) in total. However, the differences in the ME between formulas were not statistically significant. SRK-II had a significantly larger MAE than the other two formulas (*p* < 0.05). 

For the Bioline Yellow group, the SRK-II formula had the lowest ME, which was significantly lower than the Barret (*p* < 0.01) but not lower than the Haigis (*p* = 0.06). Although the MAE was also the lowest in the SRK-II formula, it was not statistically different (*p* > 0.05) from the other formulas.

For the Softec HD group, SRK-II was significantly different from Barret (*p* < 0.01) in terms of ME but was not significantly different from Haigis (*p* > 0.05). Although SKR-II had the best ME, differences in the MAE were not significant (*p* > 0.05).

In the ZCB00 group, Haigis had a lower ME than SRK-II (*p* < 0.01) but it was not significantly different from Barret (*p* = 0.09). In terms of MAE, there were no significant differences amongst the formulas (*p* > 0.05).

For the ZA9003 group, there was no statistically significant difference in the ME between the formulas. In terms of MAE, SRK-II was significantly less accurate than Barret (*p* < 0.05). No significant difference was found in the MAE between other formulas. 

Table 2 shows that the ZCB00 group had a hypermetropic shift in the ME compared to the other IOLs. 

In SRK-II, the shift in ME was significant between ZCB00 and ZA9003 (*p* < 0.05). The difference between ZCB00 and BY was borderline not significant (*p* = 0.056). In the Haigis formula, there was only significant difference in the ME when compared to BY (*p* < 0.05). In the Barret formula, the hypermetropic shift was significant (*p* < 0.01) in all IOLs except ZA9003. Differences in the MAE between ZCB00 and other IOLs were not significant (*p* > 0.05) in any of the analyzed formulas, except in the SRK-II formula when compared to BY and Softec HD (*p* < 0.01).

Table 3 shows the percentage of eyes according to the achieved postoperative ME. In the BY group, the largest portion of eyes achieved ±0.25 D by using the SRK-II formula. The Haigis formula achieved the largest portion of the eyes in all three accuracy levels (±0.25 D, ±0.5 D, ±1.0 D) in the Softec HD group. In the ZCB00 and ZA9003 group, the Barret formula had the highest percentage of eyes within ±0.25 D.

## 4. Discussion

In this study, the performance of some of the newer theoretical calculation formulas (Haigis, Barret Universal II) was compared to some of the oldest regression formulas (SRK-II) that was also used in some cases by the department. The SRK-II formula for the calculation of Bioline Yellow IOL in eyes with an axial length range from 22 to 26 mm was used. Currently, SRK-II is considered obsolete and inaccurate [9] but many eye surgical centers still use it as the only formula [10]. In this study, the different popular IOL types that are not commonly analyzed in other publications were compared. 

The mean error of prediction and the absolute value of the ME were compared. The mean error of prediction could give some information about the accuracy of the formula, but it mainly shows a systematic prediction error. If it is different from zero, it indicates that the systematic myopic or hyperopic outcomes could be predicted [11]. The MAE has been used to show the accuracy of prediction, as it is not influenced by negative values [11].

In many studies, it was shown that Barret is more accurate than the Haigis formula [8,12,13]. Similar results were observed when all the types of IOL were analyzed together. However, the difference in the ME was not statistically significant. Additionally, no constants optimizations were carried out in Haigis or any other formulas. The MAE in the Barret formula was significantly lower than in SRK-II. According to these findings, we could consider Barret as the most accurate universal formula from the analyzed formulas. These findings are confirmed by more recent studies [8,14,15,16,17]. 

If the formulas were assessed for each IOL type separately, the results were slightly different. For the Bioline Yellow group, the SRK-II formula had the lowest ME and MAE compared to the other two formulas (Table 2). The ME was significantly lower than the Barret (*p* < 0.01) but not significantly lower in Haigis (*p* = 0.06). Although the MAE was also the lowest in the SRK-II formula, it was not statistically different (*p* > 0.05) from the other formulas. This result is in contrast to the recommendations made to avoid older regression formulas [10]. If a slight myopic shift in Barret and Haigis is beneficial in monofocal lenses, it could be debatable as it could increase the functional depth of field, giving rise to more intermediate range vision. 

In the Softec HD group, SRK-II had the most accurate ME amongst the other formulas. For the Softec HD group, SRK-II was significantly different from Barret (*p* < 0.01) in terms of ME but was not significantly different from Haigis (*p* > 0.05). However, the statistically significant difference between SRK-II and Barret was probably caused by the relatively same opposite distance from zero. Although SKR-II had the lowest ME, the Haigis formulas had the lowest MAE. In this case, we could assume that the Haigis formula was the most accurate formula, but the differences in MAE were insignificant (*p* > 0.05). 

In the ZA9003 group, the formulas performance in terms of ME was similar (*p* > 0.05). In the MAE, only SRK-II performed significantly worse than the other formulas (*p* < 0.01). 

In the ZCB00 group, the ME was the lowest in the Haigis formula, which was significantly different from SRK-II (*p* < 0.01) but it was not significantly different from Barret (*p* = 0.09). In terms of the MAE, Barret was the most accurate formula but there were no significant differences amongst the formulas (*p* > 0.05). ZCB00 showed a slight hypermetropic shift in the ME, which confirms our clinical experience with this type of IOL. A hypermetropic shift was also observed by Eldaly [18]. In the SRK-II formula, the shift in ME was significant between ZCB00 and ZA9003 (*p* < 0.05). Between ZCB00 and BY, the difference was borderline not significant (*p* = 0.056). In the Haigis formula, only the significant difference in the ME was compared to BY. In the Barret formula, the difference was significant (*p* < 0.01) in all the IOLs except ZA9003. ZCB00 was the least accurate in all formulas amongst all the other IOL in terms of MAE but the differences were not significant (*p* > 0.05) in any of the analyzed formulas, except in the SRK-II formula when compared to BY and Softec HD (*p* < 0.01). 

The different results were found in Lee’s study, where ME for ZCB00 with the Haigis formula was −0.046 ± 0.46 D [19]. A PCI biometer (IOL master 500) was used in their study, but this should not cause problems in the interpretation of the results, as the IOL Master 500 and Lenstar LS900 produces very similar measurements [20]. They used A-constant personalization, which could cause better results [19]. Kim and Ha also analyzed the performance of IOL formulas in ZCB00 IOL [21,22]. In their studies, they analyzed the MAE of ZCB00 of the Haigis and Barret formula, which was lower for both formulas when compared to the results of this present study. However, they also used constants optimization.

In this study, the percentage of eyes within a certain range of prediction errors was shown in Table 3. The Barret formula achieved most of the eyes within ±0.25 D overall in all the groups combined when compared to the other two formulas. These findings are very similar to other studies [8,13,21]. Two recent formulas achieved above 85% of eyes within ±1.00 D, which is lower in other studies [8,13,21,23]. The overall score was pulled down by the ZC00 and ZA9003 groups, where all the formulas performed poorly. Surprisingly, SRK-II performed better than the recent formulas in the BY group. In all other cases, it performed the worst of all analyzed groups. The Haigis formula was the most accurate in the Softec HD group. In ZCB00 and ZA9003, Barret was the most accurate formula. However, in these two groups, especially in ZCB00, all formulas performed significantly poorer than in the other IOLs. Especially the SRK-II formula was very inaccurate. Differences in the MAE or ME for each formula in a different type of lenses could be caused by constants accuracy provided by the manufacturer.

The limitation of the study could be that surgeries were performed by three surgeons instead of only one. Although all surgeons had a similar technique, this could lead to some bias, as a result of slight interpersonal differences in surgical technique. Sample size could also be a limitation. This study compares only relatively normal eyes (with axial lengths from 21 to 26 mm), thus, the findings cannot be applied in extremely short or long eyes. Automated refractometry was measured at least two weeks after surgery. This could be one of the limitations of the study, as this could affect the effective lens position. However, changes in effective lens positions are most significant during the first postoperative week and changes in refraction are very small [24].

## 5. Conclusions

In conclusion, this study found that the Barret formula is the most accurate “universal” formula compared to SRK-II and Haigis, but different results were achieved when each IOL type was analyzed separately. We confirmed that SRK-II should not be used in most of the cases. In specific cases, SRK-II could still be a valuable formula, as shown in this study with Bioline Yellow IOL. To our knowledge, there are very few recent studies comparing IOL calculation formulas in Tecnis ZCB00 and ZA9003. Most of them are carried out on an Asian population. In this study, where no constants optimization was performed, ZCB00 showed a hypermetropic shift in ME. The performance of the analyzed formulas in ME and MAE showed that constants optimization was necessary for ZCB00 or ZA9003, either individually or by the manufacturer. In the other two analyzed types of IOL, the formulas performed comparably to other studies even without constants optimizations [8,13,21,23].

## Figures and Tables

**Table 1:** Demographic data of the sample.

		Minimum	Maximum	Mean	SD
Total (N = 120)	Age	29.00	90.00	72.78	9.31
	AL	21.35	25.99	23.59	1.18
	K	40.05	47.97	43.84	1.72
BY (N = 30)	Age	56.00	89.00	74.75	8.91
	AL	21.35	25.52	23.31	0.99
	K	40.05	47.97	43.82	1.98
Softec HDM (N = 30)	Age	62.00	86.00	77.40	5.66
	AL	21.62	25.32	23.70	0.98
	K	40.38	47.62	44.23	1.59
ZCB00 (N = 30)	Age	51.00	90.00	71.94	8.72
	AL	22.10	25.57	23.56	0.84
	K	40.85	46.49	43.94	1.50
ZA9003 (N = 30)	Age	29.00	78.00	65.54	9.64
	AL	22.20	25.99	24.62	1.42
	K	40.07	46.73	43.28	1.73

BY—Bioline Yellow. AL —Axial Length. K – Keratometry.

**Table 2:** Outcomes for each formula sorted by IOL type.

		Total (N = 120)		BY (N = 30)		Softec HD (N = 30)		ZCB00 (N = 30)		ZA9003 (N = 30)	
		Mean	SD	Mean	SD	Mean	SD	Mean	SD	Mean	SD
ME	SRK-II	0.14	0.76	0.01	0.58	0.08	0.69	0.51	0.75	−0.06	0.90
	Haigis	0.10	0.65	−0.14	0.67	0.11	0.51	0.34	0.73	0.07	0.60
	Barret	0.06	0.65	−0.20	0.59	−0.16	0.58	0.48	0.63	0.10	0.57
MAE	SRK-II	0.61	0.47	0.39	0.42	0.56	0.40	0.78	0.46	0.73	0.52
	Haigis	0.48	0.44	0.46	0.50	0.37	0.35	0.64	0.48	0.45	0.39
	Barret	0.48	0.44	0.42	0.46	0.44	0.41	0.63	0.47	0.43	0.38

ME—mean error of prediction. MAE—mean absolute error of prediction. BY—Bioline Yellow.

**Table 3:** Percentage of eyes according to achieved postoperative mean prediction error.

ME	Total (N = 120)			BY (N = 30)			Softec HD (N = 30)			ZCB00 (N = 30)			ZA9003 (N = 30)		
	SRK-II	Haigis	Barret	SRK-II	Haigis	Barret	SRK-II	Haigis	Barret	SRK-II	Haigis	Barret	SRK-II	Haigis	Barret
±0.25 D	31.7%	39.2%	40.8%	50.0%	36.7%	43.3%	30.0%	50.0%	43.3%	20.0%	30.0%	33.3%	23.3%	40.0%	43.3%
±0.50 D	49.2%	66.7%	60.8%	66.7%	76.7%	66.7%	56.7%	76.7%	63.3%	30.0%	46.7%	50.0%	43.3%	66.7%	63.3%
±1.0 D	76.7%	85.8%	85.8%	93.3%	93.3%	93.3%	86.7%	93.3%	86.7%	66.7%	73.3%	66.7%	63.3%	83.3%	83.3%

ME—mean error of prediction. BY—Bioline Yellow.

## Data Availability

The data presented in this study are available on request from the corresponding author. The data are not publicly available due to restriction in sharing of personal data.
